# Supplementary material for: Early and late adverse clinical outcomes of severe carbon monoxide intoxication: A cross-sectional retrospective study
Source: PLoS One. 2024 Aug 30;19(8):e0301399. doi: 10.1371/journal.pone.0301399 (PMC11364238; doi:10.1371/journal.pone.0301399)
Supplement: Attachment — Submitted filename: Response to reviewers.docx [file pone.0301399.s003.docx]

**Response to reviewers**

**First, we thank the reviewers for their valuable contributions.**

Reviewer #1 : Thank you for the opportunity to review your paper investigating therapies for carbon monoxide poisoning and their short and longer-term effects. The paper is well-written, making it easy to understand, despite my unfamiliarity with the topic.

-Pg 12. in your comparison with findings from Rose et al, you might consider referring to the possibility of your sample size as a reason for the inconsistent findings, not leaving this acknowledgement to the end in the Limitations.

**Response:** In the Discussion section of the article, the findings of Rose et al. are compared with those of our study, and the most important possible reason for this discrepancy is discussed in line with your suggestions.

-Pg. 12 in reporting on the results from the multivariate regression, something could be added about the importance of your finding when you state "patients who received NBOT, had at least one chronic disease, and had a higher COHb level were more than eight times more likely to develop DNS than patients without these risk factors". The "so what" statement. Also related to this statistic, please check the reporting of the related 764% comparing HBO with reference NBO, Table 5.

**Response:** We have noted the significance of this result. Furthermore, we have corrected the typo in the odds ratios in Table 5 and the accompanying text.

-Re: Data made available: I did not find the dataset, hence No for Q3

**Response:** The dataset has been added.

Reviewer #2: This paper looked at the CO poisoning and the difference in outcome depending on treatment received.

In methods:

-Is this study a prospective cohort or a cross-sectional study?

**Response:** This is a cross-sectional, observational study. We have also included this statement in the Methodology section of the study.

-Why were those below 18 years not included? seeing that consent was not a concern for data collection?

**Response:** This study was conducted in an adult emergency department. Our adult emergency unit accepts patients aged ≥ 18 years. Therefore, children were excluded from the study. There were no issues related to failure to obtain ethical consent.

-It would be better if you could include an 'operational definitions' section to define some cut off point

**Response:** 'operational definitions' section was added.

-consider debulking the tables (just include OR, Confidence interval, p-value)

**Response:** The table content has been debulked. This has been simplified in accordance with your suggestion ( Tables 4 and 5) (just include OR, Confidence interval, p-value). Tables have also been rearranged.

-In the discussion: use literature closer to the area you studied

**Response:** Dear reviewer, thank you very much for your valuable comments and suggestions. All the references used in our study are up-to-date and very comprehensive studies on the diagnosis, treatment and prognosis of CO poisoning, which are closely related to Emergency Medicine. In addition, in line with your suggestions, the discussion section of the study has been enriched with additional current and relevant sources.

-Generally revise the grammar.

**Response:** The entire article has been carefully checked again and edited for grammar and spelling.
